# IRE1α-Driven Inflammation Promotes Clearance of Citrobacter rodentium Infection

**DOI:** 10.1128/IAI.00481-21

**Published:** 2022-01-25

**Authors:** Lydia A. Sweet, Sharon K. Kuss-Duerkop, A. Marijke Keestra-Gounder

**Affiliations:** a Department of Immunology and Microbiology, University of Colorado Anschutz Medical Campus, Aurora, Colorado, USA; University of California San Diego School of Medicine

**Keywords:** *Citrobacter*, *Escherichia*, EPEC, innate immunity, ER stress, KIRA6, IRE1α, NOD1, NOD2, *Citrobacter rodentium*, IRE1a, NOD1/2

## Abstract

Endoplasmic reticulum (ER) stress is intimately linked with inflammation in response to pathogenic infections. ER stress occurs when cells experience a buildup of misfolded or unfolded protein during times of perturbation, such as infections, which facilitates the unfolded protein response (UPR). The UPR involves multiple host pathways in an attempt to reestablish homeostasis, which oftentimes leads to inflammation and cell death if unresolved. The UPR is activated to help resolve some bacterial infections, and the IRE1α pathway is especially critical in mediating inflammation. To understand the role of the IRE1α pathway of the UPR during enteric bacterial infection, we employed Citrobacter rodentium to study host-pathogen interactions in intestinal epithelial cells and the murine gastrointestinal (GI) tract. C. rodentium is an enteric mouse pathogen that is similar to the human pathogens enteropathogenic and enterohemorrhagic Escherichia coli (EPEC and EHEC, respectively), for which we have limited small-animal models. Here, we demonstrate that both C. rodentium and EPEC induced the UPR in intestinal epithelial cells. UPR induction during C. rodentium infection correlated with the onset of inflammation in bone marrow-derived macrophages (BMDMs). Our previous work implicated IRE1α and NOD1/2 in ER stress-induced inflammation, which we observed were also required for proinflammatory gene induction during C. rodentium infection. C. rodentium induced IRE1α-dependent inflammation in mice, and inhibiting IRE1α led to a dysregulated inflammatory response and delayed clearance of C. rodentium. This study demonstrates that ER stress aids inflammation and clearance of C. rodentium through a mechanism involving the IRE1α-NOD1/2 axis.

## INTRODUCTION

Citrobacter rodentium is a mouse pathogen that shares several pathogenic mechanisms with EPEC and EHEC, which are two clinically important human pathogens that infect the gastrointestinal (GI) tract (1). C. rodentium infection is initiated in the cecum and spreads to the colon, where the peak of infection is reached at 10 days postinfection (2). Over the course of infection, there is substantial damage to the epithelial barrier in the colon and the development of transmissible murine crypt hyperplasia (3). Intestinal damage caused by C. rodentium, as well as enteropathogenic and enterohemorrhagic Escherichia coli (EPEC and EHEC, respectively) in humans, is a result of the formation of attaching and effacing (A/E) lesions on the surface of intestinal epithelial cells (IECs) (3). A/E lesions are characterized by a major cellular actin rearrangement in IECs leading to the formation of pedestals and destruction of microvilli. Similar to EPEC/EHEC, C. rodentium A/E lesions are type III secretion system dependent and, thus, are driven by bacterial effector proteins (4). Given the similar pathogenic mechanisms between C. rodentium and EPEC/EHEC, understanding the murine host response to C. rodentium could lead to new insights and therapeutic interventions for the treatment of EPEC and EHEC infections in humans.

Cellular perturbations, such as those induced by pathogens, can cause endoplasmic reticulum (ER) stress. In response to this altered homeostasis, the cell activates the unfolded protein response (UPR). The UPR is mediated through three main ER transmembrane proteins, inositol-requiring enzyme-1α (IRE1α), activating transcription factor 6 (ATF6), and protein kinase R-like endoplasmic reticulum kinase (PERK) (5–7). These receptors activate transcription factors XBP1, ATF6f, and ATF4, respectively, which then bind to ER stress elements (ERSE) that result in the transcription of UPR target genes such as *Xbp1* and *Hspa5*, the gene encoding ER chaperone BiP (8, 9). *Xbp1* is a transcription factor that is upregulated by ATF6 and spliced by IRE1α (8). Spliced *Xbp1* can then upregulate *Hspa5* to help alleviate ER stress (9). Altogether, these pathways help the cell reestablish homeostasis or activate cell death if ER stress cannot be resolved. UPR activation has been shown to connect pathogen-induced ER stress to the inflammatory response during a variety of bacterial, viral, fungal, and protozoan parasite infections (10, 11). Even in the absence of infection, chemically induced ER stress promotes IRE1α activation of NOD1/2 signaling and inflammation (12). Moreover, NOD2 is important for the clearance of C. rodentium infection, but the possible connection to ER stress-induced inflammation as a response to C. rodentium infection has not been explored (13).

Here, we hypothesized that C. rodentium induces ER stress and the UPR such that IRE1α activates NOD1/2 signaling, thereby mounting an effective immune response and promoting bacterial clearance. We demonstrate that C. rodentium and a similar human pathogen, EPEC, induce ER stress *in vitro* and that the resulting inflammation is IRE1α and NOD1/2 dependent. We also show that C. rodentium induces ER stress and inflammation in the GI tract of mice and that IRE1α is required for the proinflammatory response that aids in the clearance of C. rodentium infection. Thus, ER stress mechanisms are critical for propagating inflammation and controlling C. rodentium infections.

## RESULTS

### C. rodentium induces ER stress and inflammation in mice.

**FIG 1:**
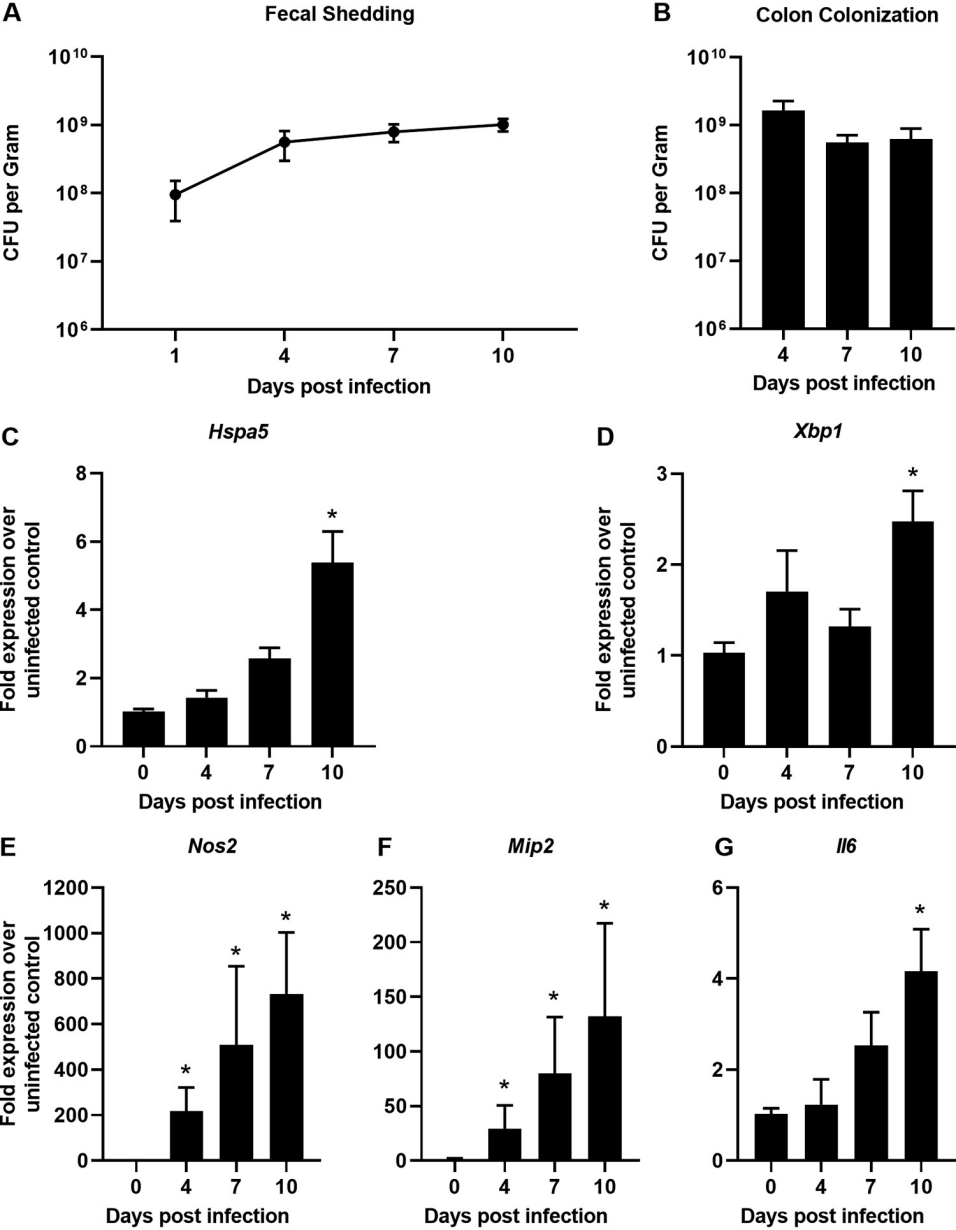
C. rodentium induces ER stress and inflammation in mice. (A) Fecal shedding of C. rodentium. (B) C. rodentium colon colonization calculated through dilution plating at the time of sacrifice for times indicated. (C to G) RNA was extracted from distal colon tissue and qRT-PCR was performed to determine the expression levels of *Hspa5* (C), *Xbp1* (D), *Nos2* (E), *Mip2* (F), and *Il6* (G) mRNAs compared to *Gapdh*. Data are shown as means ± SEM with 3 to 5 mice per group. All panels were analyzed using an ordinary one-way ANOVA followed by a Dunnett’s multiple-comparison test. *, *P* < 0.05, determined using GraphPad Prism.

To determine if C. rodentium induces ER stress *in vivo*, wild-type C57BL/6J mice were infected with C. rodentium and sacrificed at multiple days postinfection. Fecal shedding of C. rodentium was monitored for the duration of the experiment, and levels correspond to what has been previously shown in C. rodentium infection (Fig. 1A) (2). Similarly, at the time of each sacrifice C. rodentium efficiently colonized the colon, as has been reported previously (Fig. 1B). To assess the level of ER stress being induced, we examined two downstream targets of the UPR, *Hspa5* and *Xbp1*, by quantitative reverse transcription-PCR (qRT-PCR). In the colons of infected mice, *Hspa5* and *Xbp1* both reached peak upregulation at day 10 postinfection, which is around the peak of C. rodentium infection (Fig. 1C and D), according to a prior study (2). This demonstrates that the UPR is activated in C. rodentium-infected mice. At day 10 postinfection, there was also a significant upregulation of *Nos2*, *Mip2*, and *Il6* in the colons of infected mice, indicating a proinflammatory response to C. rodentium (Fig. 1E to G). There were no differences in colon colonization, which suggests that any difference in ER stress induction was not due to a greater bacterial burden (Fig. 1B). These data demonstrate that both ER stress and inflammation are induced by C. rodentium during infection in mice.

### C. rodentium and EPEC induce ER stress *in vitro*.

**FIG 2:**
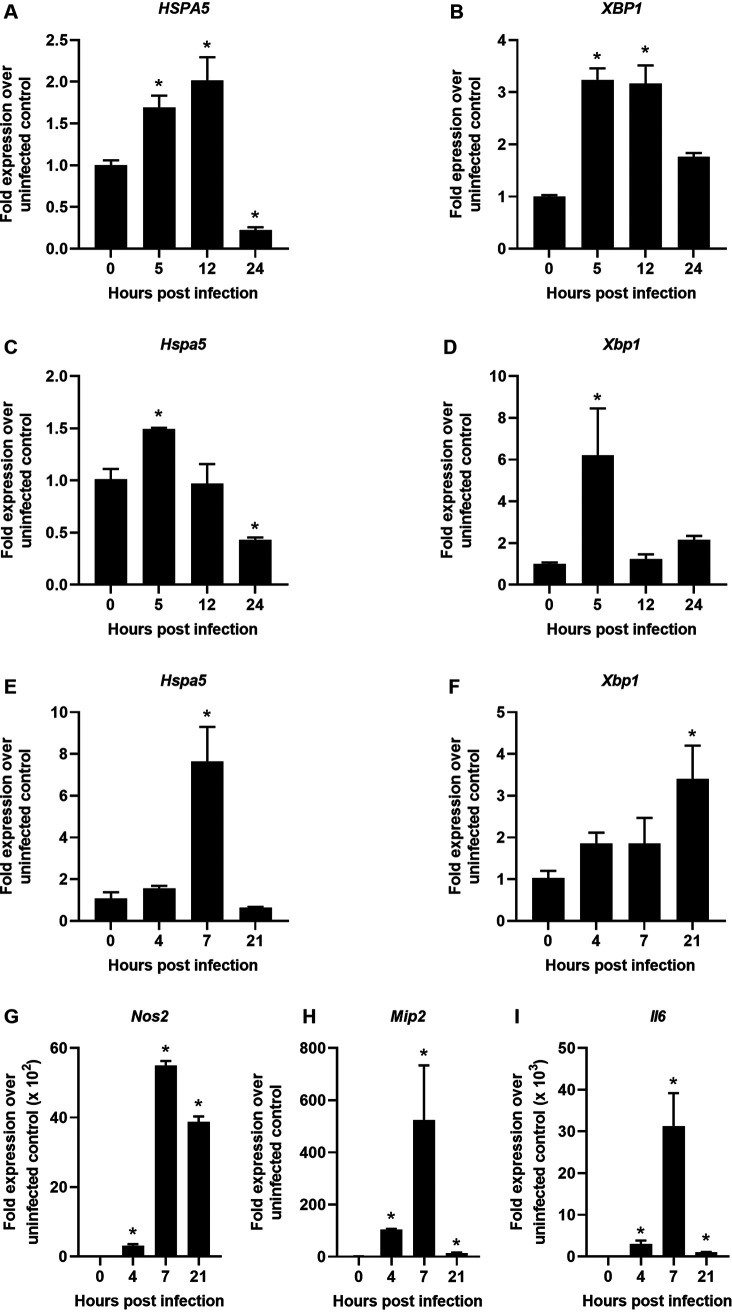
C. rodentium DBS100 and EPEC E2348/69 induce ER stress *in vitro*. (A and B) Polarized Caco-2 cells grown on transwell inserts for 7 days were infected with EPEC at an MOI of 5 for 5, 12, or 24 h. qRT-PCR was performed from cellular RNA to examine expression levels of *HSPA5* (A) and *XBP1* (B) mRNA compared to *GAPDH* expression. (C and D) MODE-K cells were infected with C. rodentium at an MOI of 10 for 5, 12, or 24 h. qRT-PCR was performed to examine expression levels of *Hspa5* (C) and *Xbp1* (D) mRNA compared to *Gapdh*. (E to I) Wild-type BMDMs were infected with C. rodentium at an MOI of 10 for 1 h, and then medium was replaced with gentamicin-containing media. Cells were incubated for an additional 3, 6, or 20 h. qRT-PCR was performed to examine expression levels of *Hspa5* (E), *Xbp1* (F), *Nos2* (G), *Mip2* (H), and *Il6* (I) compared to *Gapdh*. Data shown are from three independent experiments performed in duplicate and are presented as mean ± SEM. All panels were analyzed using an ordinary one-way ANOVA followed by a Dunnett’s multiple-comparison test. *, *P* < 0.05, determined using GraphPad Prism.

IECs are the primary site of C. rodentium and EPEC infection, so we hypothesized that they would experience UPR induction during infection. To determine the ability of EPEC to induce ER stress in human IECs, polarized Caco-2 cells were infected with EPEC and carefully monitored by microscopy for cell viability. EPEC infection upregulated *HSPA5* and *XBP1* at 5 and 12 h postinfection but by 24 h ER stress was resolved, which was not due to cell death, as monolayers were still observed by microscopy (Fig. 2A and B). These data show that EPEC induces ER stress in IECs. To model C. rodentium infection using a mouse intestinal epithelial cell line, MODE-K cells were used. At 5 h postinfection, *Hspa5* and *Xbp1* were upregulated, but ER stress was resolved quickly at 12 h postinfection (Fig. 2C and D). These data indicate that induction of ER stress is a pathogenic mechanism that is shared by C. rodentium and EPEC and that ER stress could be important for the induction of inflammation at the epithelial barrier during both infections. Thus, to examine whether ER stress corresponded to inflammation during C. rodentium infection, we infected bone marrow-derived macrophages (BMDMs). Indeed, ER stress gene expression and inflammation was evident in BMDMs (Fig. 2E to I). In BMDMs, *Hspa5* was upregulated 7 h postinfection, and *Xbp1* expression was increased throughout the time course but did not reach statistical significance until 20 h postinfection (Fig. 2E and F). These data suggest that C. rodentium can induce ER stress in macrophages as well as IECs. The peak of inflammation was 7 h postinfection, which corresponded with high levels of *Hspa5* (Fig. 2G to I). The inflammatory markers we examined are known to have a role in the host response to C. rodentium (14–18). These results suggest that ER stress induced during C. rodentium infection could drive inflammation during infection.

### ER stress-induced inflammation during C. rodentium infection is IRE1α and NOD1/NOD2 dependent.

**FIG 3:**
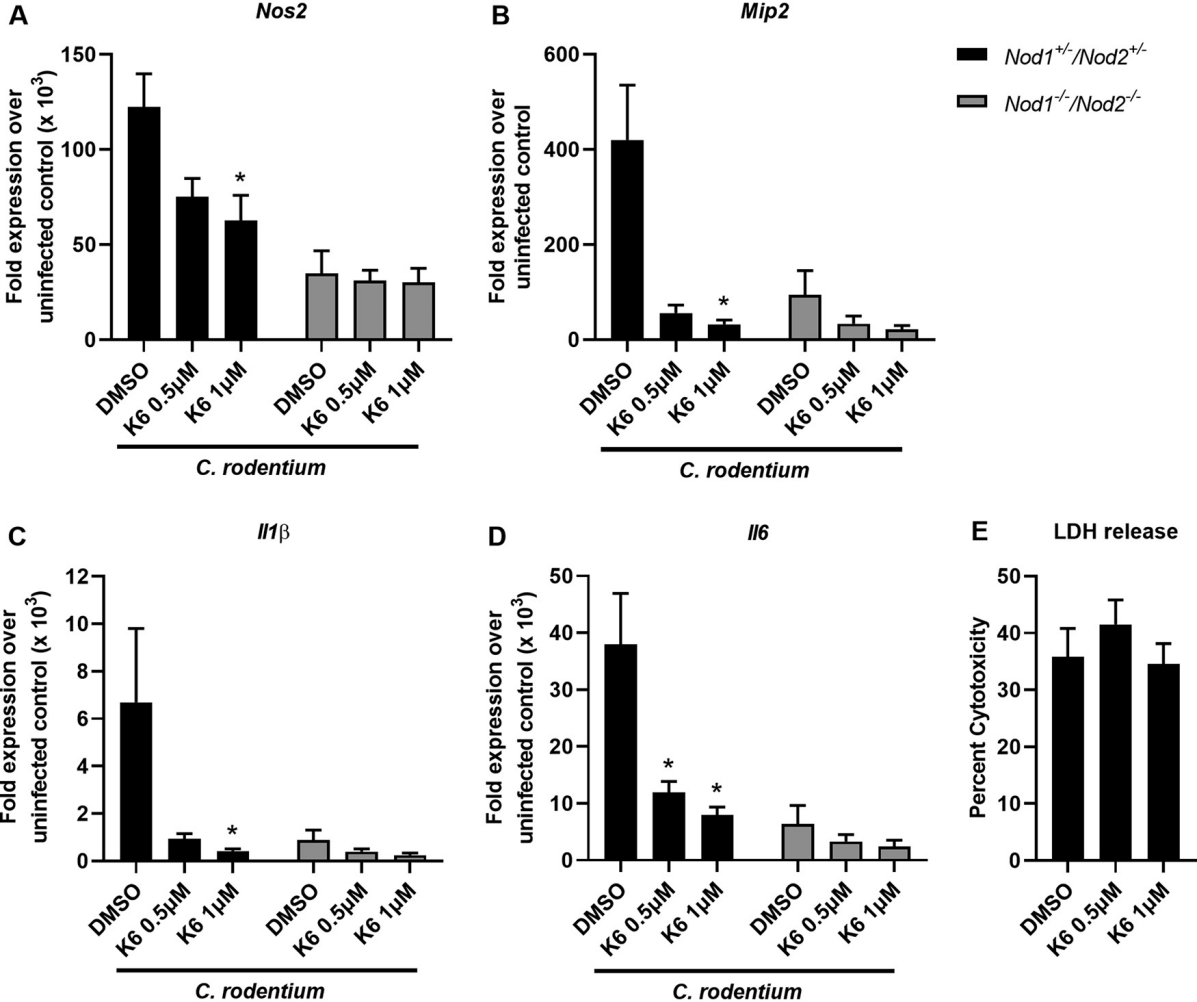
ER stress-induced inflammation during C. rodentium infection is IRE1α and NOD1/NOD2-dependent. (A to D) *Nod1^+/−^/Nod2^+/−^* and *Nod1^−/−^/Nod2^−/−^* BMDMs were pretreated with 0.5 μM or 1 μM KIRA6 or vehicle control (DMSO) for 30 min and then infected with C. rodentium at an MOI of 10 for 1 h. Medium was then replaced with gentamicin-containing medium, and cells were incubated for an additional 6 h. RNA was extracted from BMDMs, and qRT-PCR was performed to examine mRNA expression levels of *Nos2* (A), *Mip2* (B), *Il1β* (C), and *Il6* (D) compared to *Gapdh*. (E) Supernatants were used to perform an LDH release assay. Shown are data from 4 to 5 independent experiments done in duplicate. The data are presented as means ± SEM. All panels were analyzed using an ordinary one-way ANOVA followed by a Tukey’s multiple-comparison test. *, *P* < 0.05, determined using GraphPad Prism.

Prior research indicated that IRE1α can facilitate proinflammatory responses to infection and cellular perturbations (12, 19). Given that *Hspa5* and *Xbp1* transcription is downstream of IRE1α and increased during C. rodentium infection, we sought to investigate the role of IRE1α in C. rodentium-induced inflammation. Therefore, to determine whether IRE1α activation was required for inflammation during C. rodentium infection, we used an IRE1α kinase domain inhibitor, KIRA6 (12, 20). BMDMs were pretreated with vehicle or KIRA6 and then infected with C. rodentium for 1 h, washed with gentamicin, and then incubated for an additional 6 h. The time point used was based on our previous time course in BMDMs, indicating that the peak of inflammation was 7 h postinfection (Fig. 2G to I). *Nos2*, *Mip2*, *Il1β*, and *Il6* expression was significantly reduced when IRE1α was inhibited, which is consistent with prior research (Fig. 3A to D) (12). These data confirm that IRE1α is required for a strong induction of inflammation during C. rodentium infection.

IRE1α signals through NOD1/2 to activate the NF-κB pathway and downstream proinflammatory genes (12), and NOD2 activation is required for C. rodentium-induced inflammation and bacterial clearance (13). We therefore hypothesized that NOD1/2 were involved in IRE1α-mediated inflammation. To examine the role of NOD1/2 on ER stress-induced inflammation during C. rodentium infection, NOD1 and NOD2 double deficient BMDMs (*Nod1^−/−^ Nod2*^−/−^) or heterozygous controls (*Nod_1_^+/−^ Nod_2_^+/−^*) were pretreated with a vehicle control or KIRA6 and then infected with C. rodentium. While inflammation was reduced in the *Nod1^−/−^ Nod2*^−/−^ BMDMs compared to control BMDMs, as expected based on prior research (12), there was no additional decrease in inflammation when IRE1α was inhibited with KIRA6 (Fig. 3A to D). Lactate dehydrogenase (LDH) release indicates that there was no cytotoxicity when using KIRA6 at the indicated concentrations (Fig. 3E). Additionally, KIRA6 treatment in the absence of infection did not result in altered gene expression (data not shown). Together, this indicates that ER stress induced during C. rodentium infection activates IRE1α and requires NOD1/2 stimulation to ultimately lead to proinflammatory responses.

### IRE1α-dependent inflammation promotes clearance of C. rodentium infection.

**FIG 4:**
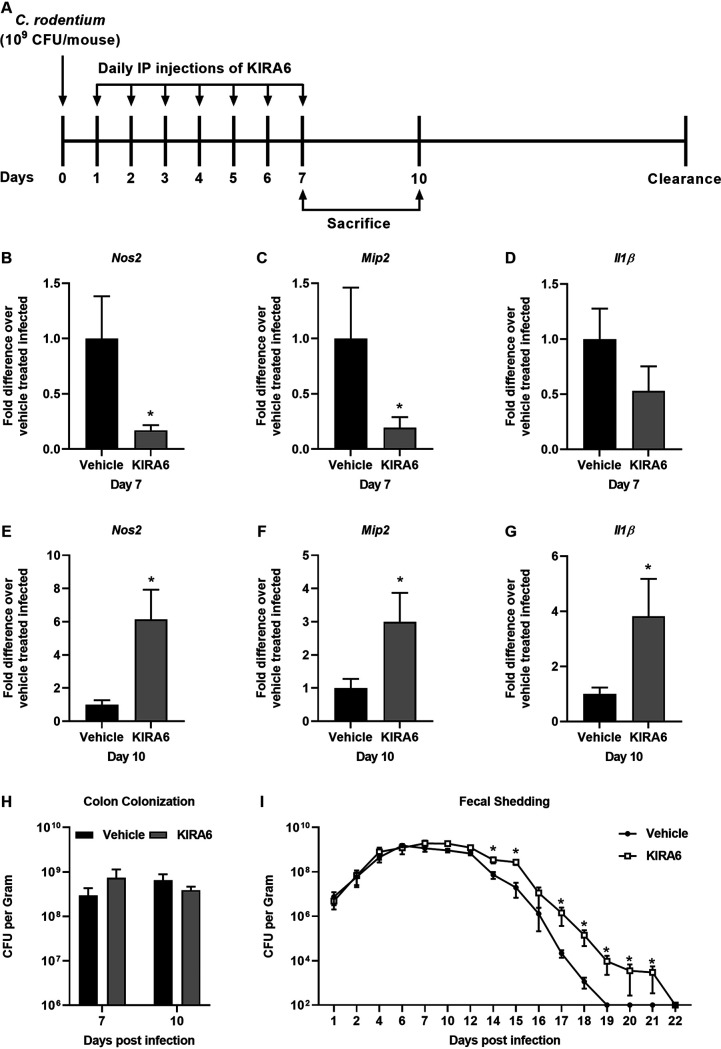
IRE1α-dependent inflammation promotes clearance of C. rodentium infection. (A) Experimental setup for KIRA6 injections *in vivo*. (B to G) Mice were infected with C. rodentium on day 0 and then injected once daily for the first 7 days postinfection. On days 7 and 10 postinfection, mice were sacrificed and RNA was extracted from the colon. qRT-PCR was performed to examine expression levels of *Nos2* (B and E), *Mip2* (C and F), and *Il1b* (D and G) of mRNA compared to *Gapdh*. The mRNA expression levels of vehicle-treated infected mice was averaged and set at 1. The mRNA expression levels of KIRA6-treated mice were compared to the average from vehicle-treated mice. (H) Colon contents collected at sacrifice were diluted and plated, and then colonies were counted. (I) Fecal pellets were collected throughout the course of infection, processed, and plated for bacterial enumeration. Five to 7 mice were used per group. The data are presented as mean ± SEM. *, *P* < 0.05 (unpaired Student’s *t* test for panels b to e and Mann-Whitney U test for panel g), determined using GraphPad Prism.

Mounting a strong immune response to C. rodentium infection is required to efficiently eradicate the bacteria (12). To determine the effect of IRE1α activation on inflammation during C. rodentium infection, mice were intraperitoneally (i.p.) injected with the IRE1α inhibitor KIRA6 for the first 7 days of infection and then either sacrificed at day 7 or 10 postinfection or maintained until C. rodentium was cleared (Fig. 4A). To assess the role of IRE1α on the inflammatory response during infection, quantitative reverse transcription-PCR (qRT-PCR) was performed on colon tissue from uninfected and infected mice treated with vehicle or KIRA6. At day 7 postinfection there was significant decreased transcription of *Nos2* and *Mip2* when IRE1α was inhibited with KIRA6 (Fig. 4B and C). This suggests that blocking IRE1α activity dampens select inflammation during C. rodentium infection. At day 10, however, we observed significant increased transcription of *Nos2*, *Mip2*, and *Il1β* in the KIRA6-treated mice, suggesting that inhibition of IRE1α results in a dysregulated immune response (Fig. 4E to G). Transcription of *Il6* was not statistically significant between KIRA6 or vehicle control treated infected groups (data not shown). C. rodentium colon colonization was not different between vehicle- and KIRA6-treated mice, indicating that the differences in inflammation are not due to differences in colonization (Fig. 4H). Altogether, these data demonstrate that IRE1α mediates inflammation during C. rodentium infection.

Since inhibition of IRE1α did not affect the ability of C. rodentium to colonize the colon up to day 10 postinfection, we sought to determine whether it would alter the overall outcome of C. rodentium persistence. To determine whether IRE1α activation altered the clearance of C. rodentium, mice were i.p. injected with vehicle or KIRA6 for the first 7 days of infection, and the infection was allowed to proceed until C. rodentium was no longer detected in the feces of infected mice. KIRA6-treated mice had higher burdens of C. rodentium starting at day 14 postinfection and maintained C. rodentium shedding in the feces significantly longer than vehicle-treated mice (Fig. 4I). These data show that activation of the IRE1α pathway is important for inflammation that aids the clearance of C. rodentium infection. Overall, we observed that ER stress is activated by C. rodentium infection to trigger the IRE1α-NOD1/2 inflammatory cascade, which, in turn, enhances clearance of C. rodentium in mice.

## DISCUSSION

Previous work showed that EHEC can induce ER stress in both monocytes and IECs, which requires Shiga toxin production (21, 22). This work highlighted the role of E. coli-induced ER stress during autophagy and cell death, but the connection of ER stress to inflammation was not examined (21, 22). However, we showed that a Shiga toxin-negative strain of EPEC can still induce ER stress, which suggests that there are Shiga toxin independent mechanisms by which E. coli can induce ER stress (Fig. 2A and B). Additional research is required to determine how a Shiga toxin-negative strain of EPEC induces ER stress and whether ER stress induction is a common pathogenic mechanism of diverse E. coli or a unique feature of EPEC and C. rodentium. C. rodentium and EPEC have a similar locus of enterocyte effacement (LEE), and it is possible that one of the proteins that is secreted into the host cell by the type III secretion system could have a role in the induction of ER stress (23). Intracellular bacteria use secretion systems to prepare the host cell for entry and these processes can trigger ER stress (24). It is therefore possible that even though C. rodentium and EPEC do not invade host cells, they still use their secretion systems to manipulate host cell processes leading to the activation of the UPR.

NOD1/2 signaling has many roles in the response to pathogens, including the stimulation of inflammatory genes through a variety of pathways (25). NOD2 was previously shown to aid in the clearance of C. rodentium by promoting recruitment of monocytes (13). *Nod2^−/−^* mice cleared C. rodentium slower and had reduced colonic inflammation at early times postinfection compared to wild-type controls (13). Our data expand the mechanism behind prior research to show that ER stress activation via IRE1α triggers inflammation and clearance of C. rodentium infection, which depends on NOD1/2 signaling. In the mice or cells treated with the IRE1α inhibitor, we saw decreased *Nos2* and *Il1β*, which are both known to be important for controlling C. rodentium infection (Fig. 3 and 4) (15, 17). We also observed decreased *Mip2* expression (Fig. 3 and 4), and when the MIP2 receptor CXCR2 was knocked out in mice, C. rodentium clearance was delayed (16). Altogether, these data show that IRE1α activation can trigger selective inflammatory pathways that impact the ability of the host to successfully and quickly clear C. rodentium infection, which is reliant on NOD1/2 signaling. This furthers our understanding of the importance and role of NOD1/2 signaling during C. rodentium infection and shows that NOD1/2 signaling can be triggered by a variety of pathogenic mechanisms, like ER stress, and not just the canonical peptidoglycan ligands (25).

Our results here expand our knowledge of pathogen-induced ER stress and inflammation, which has been shown for a variety of microbes (11). It was previously shown that by blocking IRE1α-NOD1/2 signaling, inflammation was reduced during both Brucella abortus and Chlamydia muridarum infections (12, 19). Blocking ER stress resulted in higher burdens of Chlamydia muridarum, and mice deficient in NOD1/2 cleared Chlamydia muridarum slower (19). We now show that IRE1α-NOD1/2 also has a role in inflammation and clearance in C. rodentium infection, which also may play a role in EHEC/EPEC human infections. The UPR and NOD1/2 are connected in a variety of different infections and understanding how these connections influence the host has implications outside infectious disease research. Genetic mutations in both the UPR pathway and NOD1/2 signaling have been linked to increased risk of inflammatory bowel disease (IBD), suggesting that these pathways are connected to both pathogen response and chronic inflammatory disease development (26, 27). The exact trigger of IBD is unknown, and understanding how enteric pathogens such as EPEC can impact UPR and NOD1/2 could aid in the development of treatments or preventive measures for IBD. Adherent E. coli has a role in IBD pathogenesis, but the exact mechanism is unknown and is still an area of active research (28, 29). It is possible that ER stress induction by adherent E. coli could trigger or worsen IBD in people with genetic mutations in UPR and/or NOD1/2 signaling pathways. Furthermore, many E. coli strains considered commensals may expand and invade individuals with genetic mutations in the UPR pathway or *Nod1-Nod2*, as it may be more difficult to control the commensal microbiota, and this could lead to uncontrolled inflammation and IBD flares that worsen the disease.

Altogether, we observed the importance of the UPR in controlling C. rodentium infection in mice by showing that the IRE1α arm of the UPR is critical for activating NOD1/2 signaling to elicit an inflammatory response that is important for bacterial clearance. The host intestine is constantly faced with possible pathogenic or frank pathogenic insults from the host microbiota or ingestion of enteric pathogens such as EPEC. Many mechanisms exist to control bacterial invasion, including activation of pattern recognition receptors NOD1 and NOD2 (30). Although NOD1 and NOD2 recognize and respond to bacterial peptidoglycan, we and others have shown that they also promote inflammation in response to ER stress (12, 19). Thus, it is increasingly clear that infection responses mediated by NOD1 and/or NOD2 to promote inflammation is critical to control enteric infections, such as C. rodentium. Indeed, two distinct arms of NOD1/2 activation likely exist to respond to C. rodentium infection: (i) peptidoglycan recognition and (ii) ER stress-activated IRE1α-NOD1/2 (Fig. 3 and 4). The ability of NOD1 and NOD2 to respond to ER stress further underscores the importance of NOD1 and NOD2 during infection control and the connection between ER stress and PRR responses to manage infections.

## MATERIALS AND METHODS

### Cells and bacterial strains.

MODE-K cells were propagated in Dulbecco’s modified Eagle’s medium (DMEM) (Gibco) with 1% nonessential amino acids, 2% sodium pyruvate, and 5% fetal bovine serum (FBS) (Sigma) at 37°C in 5% CO_2_ atmosphere. Caco-2 cells were cultured in Iscove's modified Dulbecco's medium (Lonza) with 1% GlutaMAX (Gibco) and 10% FBS. Bone marrow-derived macrophages (BMDMs) were differentiated from the bone marrow extracted from the tibias and femurs of *Nod1^+/−^ Nod2^+/−^* or *Nod1^−/−^ Nod2^−/−^* C57BL/6 mice bred at the University of Colorado Anschutz Medical Campus. *Nod1^−/−^ Nod2^−/−^* mice were bred with *Nod1^+/−^ Nod2^+/−^* mice to yield littermates with both *Nod1^+/−^ Nod2^+/−^* and *Nod1^−/−^ Nod2^−/−^* mice present (mouse line provided by Daniel Portnoy). BMDMs were isolated by following standard protocols. Briefly, tibias and femurs were flushed with cold RPMI to collect bone marrow stem cells, and then cells were plated in L-cell conditioned RPMI medium with 10% FBS, 1% GlutaMAX, and 1% antibiotics-antimycotics (Gibco) for 6 days to differentiate the stem cells into BMDMs (31). After differentiation, BMDMs were harvested, counted, resuspended in RPMI medium with 1% GlutaMAX and 10% FBS, and then plated for subsequent experiments. Citrobacter rodentium strain DBS100 and enteropathogenic Escherichia coli strain E2348/69 serotype O127:H6 were used for infection experiments.

### Animal experiments.

All mouse experiments were approved by the Institutional Animal Care and Use Committees at the University of Colorado Anschutz Medical Campus. Female C57BL/6 mice were purchased from The Jackson Laboratory and used for infection experiments at 6 to 8 weeks of age. Mice were infected by oral gavage with 10^9^ CFU in 100 μl of C. rodentium suspended in LB broth, Miller. Mock-infected mice received 100 μl of LB broth via oral gavage. Bacterial shedding was monitored through fecal pellet collection (2 to 3 pellets per mouse) and plated on LB plates containing nalidixic acid (Nal) at 50 μg/ml throughout the course of the infection. At 4, 7, and 10 days postinfection, mice were euthanized using CO_2_ asphyxiation followed by cervical dislocation. Colon contents were collected in PBS for plating on LB/Nal plates, and colon tissue was snap-frozen in liquid nitrogen. For KIRA6 (Sigma-Aldrich) experiments, mice were injected intraperitoneally with 200 μl of 5 mg/kg body weight of KIRA6 or vehicle control once daily for the first 7 days of infection. The vehicle control was water and DMSO at a 1:1 ratio. KIRA6 was suspended in 100% DMSO. For injections KIRA6 stock was diluted 1:1 in water and filtered. At days 7 and 10 postinfection, mice were euthanized, colon contents were collected in PBS for plating, and colon tissue was snap-frozen in liquid nitrogen. For the C. rodentium clearance experiment, fecal shedding was monitored for the duration the experiment until no colonies were present on the plates.

### Infection assays.

MODE-K cells were plated at 2 × 10^5^ in 12-well plates in 500 μl of DMEM with 1% nonessential amino acids, 2% sodium pyruvate, and 5% FBS and incubated overnight at 37°C in 5% CO_2_ atmosphere. C. rodentium strain DBS100 was grown overnight in LB at 37°C with shaking and then diluted 1:50 and grown for 3 h, with shaking at 37°C in LB broth, Miller (Fisher Scientific). Cells were infected the day after plating with C. rodentium at a multiplicity of infection (MOI) of 10 for 5, 12, or 24 h.

Caco-2 cells were plated at 2.5 × 10^5^ in 500 μL of Iscove’s modified Dulbecco’s medium with 1% GlutaMAX and 10% FBS on 12-well transwell inserts (Greiner Bio-One) and were allowed to polarize for 7 days at 37°C in 5% CO_2_ atmosphere. Polarization was confirmed by monitoring transepithelial electrical resistance (TEER) with an EVOM2 (World Precision Instruments) until the TEER stabilized. EPEC strain E2348/69 serotype O127:H6 was grown overnight and diluted 1:50 and grown for 3 h at 37°C, with shaking. Caco-2 cells were then infected with EPEC at an MOI of 5 and incubated for 5, 12, or 24 h postinfection.

BMDMs were plated at 2.5 × 10^6^ in 1 ml of RPMI medium with 1% GlutaMAX and 10% FBS on 6-well plates and incubated for 48 h. BMDMs were pretreated for 30 min with 0.5 μM or 1 μM KIRA6 or vehicle control (0.01% DMSO). Next, BMDMs were infected at an MOI of 10 and incubated for 1 h. After 1 h, medium was replaced with RPMI containing 1% GlutaMAX, 10% FBS, gentamicin (50 μg/ml), and KIRA6 or vehicle control, and cells were incubated for an additional 3, 6, or 20 h at 37°C in 5% CO_2_ atmosphere.

### qRT-PCR.

RNA was isolated from MODE-K cells, Caco-2 cells, BMDMs, and colon tissue using TRI Reagent (Molecular Research Center) according to the manufacturer’s instructions. Reverse transcription was performed using 1 μg of DNase-treated RNA (TURBO DNA-free kit) with TaqMan reverse transcription reagents (Applied Biosystems). Real-time PCR was performed using SYBR green PCR master mix (Applied Biosystems) and the Quantstudio 7 Flex real-time PCR system (Applied Biosystems). Fold change in mRNA levels was calculated using the delta-delta comparative threshold cycle (*C_T_*) method. All targets were normalized to expression levels of *Gapdh* or *GAPDH* (Table 1).

**TABLE 1:** Primer sequences used for qRT-PCR

Target	Forward sequence	Reverse sequence
*hGAPDH*	CCAGGAAATGAGCTTGACAAAGT	CCCACTCCTCCACCTTTGAC
*hHSPA5*	CGAGGAGGAGGACAAGAAGG	CACCTTGAACGGCAAGAACT
*hXBP1*	TGCTGAGTCCGCAGCAGGTG	GCTGGCAGGCTCTGGGGAAG
*mGapdh*	TGTAGACCATGTAGTTGAGGTCA	AGGTCGGTGTGAACGGATTTG
*mHspa5*	GAGCGTCTGATTGGCGATGC	TTCCAAGTGCGTCCGATGAGG
*mXbp1*	GAGTCCGCAGCAGGTG	GTGTCAGAGTCCATGGGA
*mNos2*	TTGGGTCTTGTTCACTCCACGG	CCTCTTTCAGGTCACTTTGGTAGG
*mMip2*	AGTGAACTGCGCTGTCAATGC	AGGCAAACTTTTTGACCGCC
*mIl6*	TCCAATGCTCTCCTAACAGATAAG	CAAGATGAATTGGATGGTCTTG
*mIl1β*	CCTGAACTCAACTGTGAAATGCC	TCTTTTGGGGTCCGTCAACTTC

### LDH release assay.

Supernatants from BMDMs were harvested and used for an LDH release assay. CytoTox 96 nonradioactive cytotoxicity assay (Promega) was used to determine LDH release. A 10× lysis solution from the kit was added to control wells 45 min before collection of supernatants for maximum LDH release reading; 50 μl of CytoTox96 reagent was added to 50 μl of sample supernatant and then incubated in the dark for 30 min. Next, 50 μl of stop solution was added to each well, and then the plate was read at 490 nm on a Tecan Infinite 200 PRO plate reader. Percent cytotoxicity was calculated as (experimental OD_490_)/(maximum LDH release OD_490_) × 100.

### Statistical analysis.

Data analysis was performed with GraphPad Prism. Data are shown as mean ± standard error of the mean (SEM). One-way analyses of variance (ANOVAs), Student’s *t* tests, and Mann-Whitney U tests were performed. Outliers were identified in GraphPad Prism using the ROUT method.
